# Angiogenesis and Breast Cancer

**DOI:** 10.1155/2010/576384

**Published:** 2010-10-07

**Authors:** Adhemar Longatto Filho, José Manuel Lopes, Fernando C. Schmitt

**Affiliations:** ^1^Laboratory of Medical Investigation (LIM), School of Medicine, University of São Paulo, 01246-903 São Paulo-SP, Brazil; ^2^School of Health Sciences, Life and Health Sciences Research Institute, University of Minho, Campos of Gualtar, 4710-057 Braga, Portugal; ^3^Institute of Molecular Pathology and Immunology, University of Porto, IPATIMUP, Rua Roberto Frias s/n, 4200 Porto, Portugal; ^4^Medical Faculty, Porto University, 4099-002 Porto, Portugal

## Abstract

Angiogenesis is an essential step for breast cancer progression and dissemination. The development of new blood vessels in cancer setting (angiogenesis) is conducted by numerous physiological and pathological stimuli, where the main stimulus is hypoxia. The knowledge of different molecular pathways regulating angiogenesis is constantly growing. An increased and complex scenario of angiogenesis is nowadays available in breast cancer, specifically, and permits not only to understand most of the important phases of neoplastic growth but also offer an exciting perspective for new therapeutic proposals based on blocking new blood vessels sprouting. This review focused on historical and recent understanding of angiogenesis occurrence in breast cancer.

## 1. Introduction

The association of angiogenesis and cancer has been credited to the visionary pioneer Judah Folkman (1933–2008), who firstly stated that tumour growing was directly dependent the blood vessel network development [1]. The discovery of angiogenic molecules at earlier seventh's, prompt stimulated several works addressed to answer a number of questions related to the cancer development and regulation dependent on blood vessels vascularisation.

Angiogenesis is a central part of many normal homeostatic processes and nonneoplastic diseases. Regarding malignant neoplasia, it is now evident that tumours have a very limited capacity to grow without vascular support; therefore, formation of blood vasculature is obligatory step to sustain the influx of essential nutrients to the cancer mass. Blood neovascularisation is a complex phenomenon that involves several molecular players and cells. Interaction between stromal and epithelial components is importantly enhanced, and most of the events observed in wound repair are maintained [2]. 

Some previous historical observations credited to Folkman and colleagues already figured out the crucial role of angiogenesis in cancer setting [1]. The observation that the tumour growing largely depends on angiogenic sprout, indeed, has been studied for more than six decades in several* in vivo* models [3], and the maximum values of 1 to 2 mm were recognized as the limit for neoplastic expansion without new blood vessels formation [1]. 

Molecular players of angiogenesis have been characterized since the early years of angiogenic studies, and one of the most prominent stimulating growing factors is certainly the vascular endothelial growth factor family. The most prominent member of this family, vascular endothelial growth factor (VEGF, VEGF-A) is the foremost controller of physiological and pathological angiogenesis. Accordingly, numerous VEGF inhibitors have been approved by the North American Food and Drug Administration (FDA) for the treatment of advanced cancer and neovascularisation related to the macular degeneration [4]. 

There are several molecules and signalling pathways that drive the new formation and assembly of blood vessels. Further than the well-known angiogenic factors and their receptors, such as VEGF and its receptors (VEGFR), Angiopoietin-Tie, Ephrin-EphRs, and Delta-Notch that play the major regulator processes of angiogenesis in humans [5], there are also many other molecules directly or indirectly related to the new vessels sprout, which include Fibroblast Growth Factor (FGF) and Thrombin receptors among others [6]. The consequence of so many physiologic and pathologic options to the occurrence of blood vessels sprout is the obvious consideration to create a plethora of antagonists that should be able to block the angiogenic growth, which is received from oncologists enthusiastic support to treat breast cancer [7]. This is important because angiogenic activity has been shown to be crucial to breast cancer progression. Therefore, the blockage of VEGF action is supposed to be a very promising therapeutic alternative, mainly if associated to the ordinary chemotherapy. Nevertheless, all results until now reported are, indeed, incipient, which maintain the motivation for further investigation to a more comprehensive understanding of the accurate role of anti-VEGF therapy [7]. 

**Figure 1:**
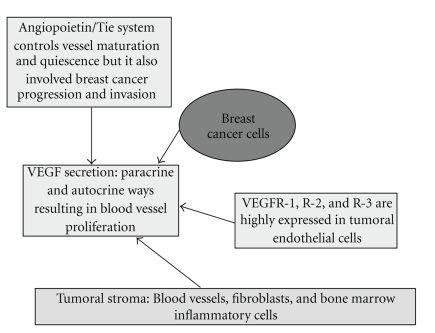
Schematic representation of molecular players involved in paracrine and autocrine VEGF secretion. Tumour cells are the major source of VEGF production, but alternative cells are currently credited as important sources to release VEGF. VEGF receptors expressed in endothelial cells have pivotal role in cancer angiogenesis and angiopoietin 1, and Tie2 receptor support the new vessel stabilization, whereas Ang-2 is thought to antagonize the stabilizing action of Ang-1. Tie-2 receptor was recently recognised to critically participate on breast cancer development and bone metastasis.

Figure 1 resumes the role of the principal molecular players involved with breast cancer progression. Block of the pathways that drive these molecular signalling is the rationale basis to anti-angiogenic therapies. Antiangiogenic therapy is a very exciting topic of the modern oncology because most of the angiogenic ligands and receptors are functionally active in tumour mass progression and can share some combinative actions with lymphatic vessels growth. Consequently, the rationale for anti-angiogenic therapy can also favour the obstruction of lymphatic vessels development, which potentially hampers the metastatic budding of the tumors [8].

Due to the complexity of neovascularisation phenomena in cancer scenario, this paper will highlight the main courses of angiogenesis studies related to the breast cancer, and describe the principal findings in four areas: experimental studies with VEGF expression in breast cancer (that include fundamental information about the importance of VEGF family in breast cancer development); the meaning of blood microvessel density that embrace diagnostic/prognostic parameters of angiogenesis; the role of angiopoietins and Tie-2 receptor in breast cancer angiogenesis and clinical approach of anti-angiogenic therapies. Experimental studies of VEGF expression in breast cancer development will introduce the theme, in order to report important statements that support angiogenesis studies in cancer; secondly, the value of blood vessel density (BVD) assessment will be discussed because there is a significant correlation between high BVD and worse prognosis in many, but not all, cancers; also, there are disputable data related to the BVD meaning in breast cancer behaviour. The final part will be dedicated to the role of angiopoietins and Tie-2 receptor in breast cancer angiogenesis because there are preclinical evidence that these receptors can directly influence the blood vessel sprout in breast cancer and also be implicated as a potential therapeutic target. This section is a connection to link the two previous themes and the final section, which will explore the clinical evidence of anti-angiogenic therapies.

## 2. Experimental Studies with VEGF Expression in Breast Cancer

The role of VEGF has been intensely tested in pre-clinical conditions to support the introduction of anti-angiogenic drugs in the clinical setting. VEGF and its receptors have been intensively studied in cultured cells in order to establish the algorithms to be tested in breast cancer therapy. Recent experimental results have endorsed the premises that angiogenic sprout in solid tumours, particularly in breast carcinomas, is regulated not only by VEGFR-2, a VEGF receptor, but also by VEGFR-3, a VEGFC lymphangiogenic receptor that is importantly expressed in tumour mass mediating blood vessel proliferation [9]. These pre-clinical assays have showed that the overexpression of VEGFR-2 and VEGFR-3 is found in both blood and lymphatic conduits, which imply that the major clinical mechanism of action of VEGF signalling inhibitors probably occurs more importantly in tumour vessels rather than tumour cells. Additionally, the upregulation of VEGFR-3 observed in cancer blood vessels point out the possibility to add in anti-angiogenic therapy the dual combination of VEGFR-2/VEGFR-3 target [9].

In clinical settings, for example, anti-VEGF antibody bevacizumab has been tested as adjuvant therapy, maintenance therapy, or in combination with both chemotherapy and other targeted agents such as the epidermal growth factor receptor kinase inhibitor erlotinib. Moreover, ramucirumab and IMC-18F1, monoclonal antibodies that target the VEGF receptors VEGFR-2 and VEGFR-1, have been also tested as well as the aflibercept, a peptide-antibody fusion targeting VEGF ligand. Presently, its recognized that is essential to targeting other angiogenic signalling pathways such as platelet-derived growth factor-C (PDGF-C), *bombina variegata* peptide 8 (Bv8, also known as prokineticin-2), and VEGFR-3, which might enhance the therapeutic response in anti-VEGF resistant tumors [8]. 

A very recent and challenging novelty concerned the current knowledge about VEGF family. Nowadays it is known that VEGF-A has alternatively spliced isoforms that inhibit neovascularisation and tumour growth [10]. Interestingly, the acidic microenvironment changes are responsible for the VEGF alternative splicing.

Furthermore, it was also reported that the existence of a splice variant of the gene encoding vascular endothelial growth factor receptor-2 (VEGFR-2) that encodes a soluble protein nominated VEGFR-2 (sVEGFR-2), inhibits lymphangiogenesis, but not angiogenesis, by blocking VEGF-C function. Thus, the modulation of VEGFR-2 might have therapeutic effects in treating tumour lymphangiogenesis among other diseases related to the lymphatic proliferation [12]. Additionally, in vitro model using MCF-7 cells has shown that VEGFR2 repression is supposed to be also related to 17 *β*-Estradiol (E2) activity [13]. Indeed, recent evidence has shown that VEGFR2 expression in MDA-MB-231 and MCF-7 breast cancer cells is low, whilst VEGFR1 expression is constantly abundant, and NRP1 expression is variable. VEGFR1 expression knockdown by siRNA (siVEGFR1) significantly decreased the survival of breast cancer cells through downregulation of protein kinase B (AKT) phosphorylation, although VEGFR2 or NRP1 knockdown has no effect on the survival of these cancer cells [14]. 

VEGF expression in normal glandular structures is assumed to be constantly lower than in breast lesions, with the highest expression in ductal tumours when compared with lobular lesions. However, there is no clear evidence that VEGF expression correlates with the microvascular density [15]. This is interesting because no significant differences have been reported between vascular densities of the two types of invasive carcinoma, although VEGF protein and VEGF mRNA expressions are significantly superior in invasive ductal than in invasive lobular carcinoma, which suggest that VEGF is important in angiogenesis in invasive ductal carcinoma, but that other angiogenic factors are essential in invasive lobular carcinoma angiogenesis [16].

Recent experimental data suggested an intrinsic relationship between hormonal status of breast tumour cells and angiogenesis. VEGF released by activated stroma, for example, increases the growth of ER-positive malignant epithelial cells and of adjacent normal epithelium. Interestingly, the alteration of the phenotype of breast cancers from oestrogen-dependent to oestrogen-independent growth is associated to the failure of antiestrogenic tumour therapies [17]. Furthermore, the overexpression of VEGF by oestrogen-dependent MCF-7 breast cancer cultured cells could hamper estrogen-dependent tumour growth in mice submitted to the ovarian ablation [18]. Finally, mutations in BRCA1, via their interaction with ER-*α*, promote carcinogenesis through the hormonal regulation of mammary epithelial cell proliferation and affect the regulation of VEGF function, which may lead to cancer growth and angiogenesis [19].

## 3. The Meaning of Blood Microvessel Density

The real importance of blood microvessel density (MVD) is still controversial. Most of the available data have same degree of discrepancy related to the significant correlation between high MVD and poor breast cancer prognosis [20]. Impressive promising data emerged with Folkman's findings, suggesting the MVD assessment as an independent predictor of metastatic disease either in axillary lymph nodes or at distant sites (or even both). Therefore, the evaluation of breast cancer MVD was assumed to select patients with early breast carcinoma for aggressive therapy [21]. 

Data emerged from studies that highlighted the blood MVD as a prognostic factor to breast cancer was initially accepted as a powerful parameter to identify the more aggressive phenotypes of breast cancer [21]. However these initial results were not confirmed and different findings obligate the revision of primary concepts [20]. The new vessels developed in tumor setting are not adequately assembled and these fragile conduits are demonstrated to be collapsed in intratumour mass. Accordingly, the intra-tumour blood vessels newly formed are faint or even not functional [6].

Presently, the assessment of MVD by the blood and lymphatic markers is credited to be a significant unfavourable prognostic factor for long-term survival in breast cancer [6, 22] besides being a likely therapeutic target for anti-angiogenic therapy. However, there are no robust evidences yet to ascertain how important it is to block lymphangiogenesis activity to prevent lymphatic spread [23]. The most intriguing finding recently reported is the participation of the most powerful angiogenic growth factor, VEGF-A, in neoplastic lymphangiogenesis as well. Also, the growth of the lymphatic vasculature in the sentinel lymph node is initiated before cancer cells arrived at these loci, suggesting that VEGF-A (and also VEGF-C, a specific lymphangiogenic factor) secreted by the tumour cells is drained to the lymph nodes, inducing lymphangiogenesis there [23].

There are important changes occurring in the neoplastic microenvironment during the different morphological alterations of hyperplasic and pre-invasive breast lesions. Interestingly, angiogenesis is observed before any significant alteration in tumour microenvironment in preinvasive breast lesions. A phenotype combination characterized by highly expressed VEGF in epithelial cancer cells and smooth muscle actin positive/CD34 negative reaction in stromal cells is predominantly identified in intermediate and high grade ductal carcinoma in situ (DCIS). Altogether, these findings are supposed to predict the progression of DCIS to invasive carcinoma, and also helpful to plan therapeutic strategies using both anti-angiogenic factors and factors that selectively target the components of tumour stroma [24]. This is important because the expression of endothelin (ET)-1, a vasoactive peptide primarily produced in endothelial, vascular smooth muscle, and epithelial cells that have been demonstrated significantly increased in numerous human malignancies including breast cancer. ET-1 and its receptors (ETAR and ETBR) increased expressions are associated with increased VEGF expression and higher MVD of breast carcinomas, suggesting that ET-1 and its receptors are involved in the regulation of breast cancer angiogenesis [25]. In addition, increased expression of ETAR in breast carcinomas is associated with resistance to chemotherapy, which indicates that the determination of ETAR status should be used as predictive marker for identifying patients less likely to be responsive to conventional chemotherapy [26]. 

Tumour microenvironment is also the scenario for the enhanced infiltration of tumour-associated macrophage (TAM) that is significantly associated with both the high VEGF expression and high MVD, which advocate a prognostic relation of TAM infiltration and tumour angiogenesis [27, 28]. Both MVD and VEGF expression are significantly correlated with tumour grade and lymph-node invasion, and TAMs correlates with mitotic activity index in ductal breast carcinoma [24]. However, augmented expression of VEGF and high MVD were not found associated with the lobular carcinoma prognosis [29].

Pigment epithelium-derived factor (PEDF), is a secreted glycoprotein recognized to be important to angiogenesis inhibition. The specific mechanism by which PEDF acts is still obscure, but PEDF is currently considered as a candidate antitumor agent. Decreased intra-tumour expression of PEDF is associated with a higher microvessel density (MVD) and poorer clinical outcome. Low PEDF expression significantly correlate with higher MVD and is assumed as an independent prognostic factor [30].

## 4. The Role of Angiopoietins and Tie-2 Receptor in Breast Cancer Angiogenesis

Angiogenesis development also involves the participation of angiopoietin (Ang-1 and Ang-2), an endothelial growth factor found to be a ligand for the endothelium-specific tyrosine kinase receptor Tie-2. Ang-1 is recognized to play an essential role in maintaining and stabilizing mature vessels by promoting the interaction between endothelial cells and surrounding support cells, whereas Ang-2 is thought to antagonize the stabilizing action of Ang-1. Under malignancies, Ang-1 and Ang-2 expressions are both elevated in tumour cells, whereas the Ang-2 expression is more commonly upregulated than Ang-1 or Tie2 expressions. The ratio of Ang-1 and Ang-2 expressions clearly favours Ang-2 that appears to be significantly associated with angiogenesis in the tumour tissues [31]. Not surprisingly, the Ang-2 expression was demonstrated to be closely correlated with VEGF expression and MVD in breast cancer as well; high MVD has been frequently found in invasive ductal carcinoma of the breast with a high expression of VEGF and Ang-2. Altogether, these markers are also supposed to show a major correlation with poor survival rates that therefore represent strong prognostic impact in breast cancer [32].

A new function of Tie2 in osteoclastogenesis and osteolytic bone invasion of breast cancer was recently reported. Tie-2 receptor has a critical participation on breast cancer development related to the bone metastasis frequently associated to the breast cancer progression. The expression of Tie2 is considerably increased in human breast cancer tissues as compared with normal and benign breast tumours and it is also present in hematopoietic stem/precursor cells. Evidence that genetic deletion of Tie2 (or neutralization) importantly impaired osteoclastogenesis in an embryonic stem cell model has emerged. Conversely, deletion of Tie2 has no effect on osteoblastogenesis. Neutralization of Tie2 activity in vivo significantly inhibited osteolytic bone invasion and tumour development in a mammary tumour model that correlates with reduction of osteoclasts and tumour angiogenesis. Importantly, Tie2 was also indentified as a therapeutic target for controlling the tumour angiogenesis and as well as the osteolytic bone metastasis in breast cancer [33].

## 5. Clinical Approach of Antiangiogenic Therapies

Breast cancer is paramount in terms of specific targets for cancer therapy. A number of novelties have emerged since the promising introduction of trastuzumab, an antiepidermal growth factor receptor (HER) used worldwide. Currently, there are different options for specific therapies that include monoclonal antibodies such as pertuzumab that bind to receptors on the cell surface, and tyrosine kinase inhibitors such as lapatinib, which target intracellular pathways as the epidermal growth factor receptor. Combination with different targets became common. These comprise the monoclonal antibody bevacizumab, which blocks the activity of VEGF receptor, and multitargeted tyrosine kinase receptors with anti-angiogenic and antiproliferative activities, for instance sunitinib. The antibodies combination targeting both the HER family and angiogenic pathways (e.g., trastuzumab plus bevacizumab) is also valuable for clinical setting [34]. Despite of hopeful evidence, judicious evaluation to indicate specific treatment should be taken because most of the breast cancers have a particular phenotype that can preclude the efficiency of monoclonal therapy. Bevacizumab, for example, is highly recommended for triple negative phenotype highly proliferative tumors, with enhanced angiogenesis that supports rapid growth and early metastases and have been found to have high levels of VEGF [35]. However, the breast cancer MVD assessed by endoglin (CD105) does not help to indicate the histopathologic phenotype prone to be referred to anti-angiogenic therapy [36]. Interestingly, endoglin (CD105), a coreceptor in the TGF-beta receptor complex, is believed to be a useful target for anti-angiogenic therapy. Endoglin vaccine activates the antigen-presenting dendritic cells, mediated by CD8+ T cells against endoglin-positive target cells. Curative vaccine may contribute to breast cancer therapy [35]. Similarly, a DNA vaccine against murine transcription factor Fos-related antigen 1, which is overexpressed in aggressively proliferating D2F2 murine breast carcinoma have been used against breast cancer development and metastatic progression combining the action of immune effector cells with suppression of tumor angiogenesis [37].

The use of minigene vaccines has grown and is competing with antimonoclonal therapies. Recently, a VEGFR-2 vaccine was successfully tested in animal model. An oral minigene DNA vaccine against murine vascular endothelial growth factor receptor-2 (FLK-1), the most important receptor in angiogenesis, protected against tumors of different origin in syngeneic BALB/c mice. Notably, the minigene vaccine has similar efficacy as a vaccine encoding the whole FLK-1 gene [38]. 

The translational approach between experimental and clinical treatment based on some innovative anticancer therapies require robust evidence for cancer reduction and metastasis prevention without major drug toxicities. Basically, breast cancer is a heterogeneous disease with different molecular players of the cell-matrix cross-talk that regulate growth, survival and, consequently, response to therapy. Therefore, the management of this tumor involve an ample comprehension of breast cancer heterogeneity, the biological nature of any given tumor as well the existence of better personalized management options [38] that take in account mechanisms of inherent/acquired resistance to cancer treatment.

Potentially, all promoters of angiogenesis could be blocked by a specific anti-angiogenic therapy. Hypoxia-inducible factor-1 production, for example, that leads to an augmented VEGF transcription is a primordial target to avoid angiogenesis. However, the blockage of promoter factors involves a complex and redundant mechanisms of angiogenesis that are difficult to be fully understood. Moreover, hypoxia can also induce the overexpression of other pro-angiogenic molecules such as nitric oxide synthase, platelet-derived growth factor (PDGF), transforming growth factors alpha and beta, basic fibroblastic growth factor (bFGF), and a class of protein growth factors called the angiopoietins [11]. The blockage could be also considered for specific receptors of angiogenesis stimulation. Most of the recognized receptors are receptor tyrosine kinases (RTKs) present on the surface of different types of cells. These include PDGF receptors (PDGFR*α* and PDGFR*β*), VEGF receptors (VEGFR1, VEGFR2 and VEGFR3), stem cell factor receptor (KIT), Fms-like tyrosine kinase 3 (FLT3), colony stimulating factor receptor type 1 (CSF-1R) and the glial cell-line-derived neurotrophic factor receptor RET [11]. Table 1 summarizes the principal inhibitors of angiogenesis currently used.

## Figures and Tables

**Table 1:** Inhibitors of angiogenesis currently used in clinical practice [11].

Angiogenic inhibitor	Target	Current clinical use
Bevacizumab	VEGF	First approved by the United States Food and Drug Administration (FDA) in February 2004 for patients with metastatic colorectal cancer. Nowadays it is used for other malignancies, including breast cancer.
Sorafenib	VEGFR2 and 3, PDGFR-*β*, FLT3 and KIT	Renal cell carcinoma, Hepatocellular carcinoma,
Sunitinib	PDGFR*α*, PDGFR*β*, VEGFR1, VEGFR2, VEGFR3, KIT, FLT3, CSF-1R, and RET	Renal cell carcinoma, Gastrointestinal stromal tumors
Thalidomide	fibroblast growth factor-2 (FGF-2) and tyrosine kinase FGF receptors	Myeloma
Aflibercept (Soluble VEGF receptors)	VEGF-A, VEGF-B, and placental growth factor (PlGF)	Metastatic nonsmall cell lung cancer, Prostate cancer
Vascular disrupting agents (VDAs)	The biological VDAs combine an endothelium-targeting molecule with a toxin or pro-coagulant	Anaplastic thyroid cancer

## References

[1] Folkman J, Merler E, Abernathy C, Williams G (1971). Isolation of a tumor factor responsible or angiogenesis. *Journal of Experimental Medicine*.

[2] Kerbel RS (2008). Tumor angiogenesis. *The New England Journal of Medicine*.

[3] Algire GH, Chalkley HW, Legallais FY, Park HD (1945). Vascular reactions to normal and malignant tissue in vivo. I vascular reactions of mice to wound and to normal and neoplastic transplants. *Journal of the National Cancer Institute*.

[4] Ferrara N (2009). Vascular endothelial growth factor. *Arteriosclerosis, Thrombosis, and Vascular Biology*.

[5] Shibuya M (2008). Vascular endothelial growth factor-dependent and -independent regulation of angiogenesis. *Journal of Biochemistry and Molecular Biology*.

[6] Duarte M, Longatto Filho A, Schmitt FC (2007). Angiogenesis, haemostasis and cancer: new paradigms and old concerns. *Jornal Brasileiro de Patologia e Medicina Laboratorial*.

[7] Bando H (2007). Vascular endothelial growth factor and bevacitumab in breast cancer. *Breast Cancer*.

[8] Hsu JY, Wakelee HA (2009). Monoclonal antibodies targeting vascular endothelial growth factor: current status and future challenges in cancer therapy. *BioDrugs*.

[9] Smith NR, Baker D, James NH (2010). Vascular endothelial growth factor receptors VEGFR-2 and VEGFR-3 are localized primarily to the vasculature in human primary solid cancers. *Clinical Cancer Research*.

[10] Rennel ES, Varey AHR, Churchill AJ (2009). VEGF_121_b, a new member of the VEGF_xxx_b family of VEGF-A splice isoforms, inhibits neovascularisation and tumour growth in vivo. *British Journal of Cancer*.

[11] Eichholz A, Merchant S, Gaya AM (2010). Anti-angiogenesis therapies: their potential in cancer management. *OncoTargets and Therapy*.

[12] Albuquerque RJC, Hayashi T, Cho WG (2009). Alternatively spliced vascular endothelial growth factor receptor-2 is an essential endogenous inhibitor of lymphatic vessel growth. *Nature Medicine*.

[13] Higgins KJ, Liu S, Abdelrahim M (2008). Vascular endothelial growth factor receptor-2 expression is down-regulated by 17*β*-estradiol in MCF-7 breast cancer cells by estrogen receptor *α*/Sp proteins. *Molecular Endocrinology*.

[14] Lee TH, Seng S, Sekine M (2007). Vascular endothelial growth factor mediates intracrine survival in human breast carcinoma cells through internally expressed VEGFR1/FLT1. *PLoS Medicine*.

[15] Viacava P, Naccarato AG, Bocci G (2004). Angiogenesis and VEGF expression in pre-invasive lesions of the human breast. *Journal of Pathology*.

[16] Lee AHS, Dublin EA, Bobrow LG, Poulsom R (1998). Invasive lobular and invasive ductal carcinoma of the breast show distinct patterns of vascular endothelial growth factor expression and angiogenesis. *Journal of Pathology*.

[17] Kawai H, Li H, Chun P, Avraham S, Avraham HK (2002). Direct interaction between BRCA1 and the estrogen receptor regulates vascular endothelial growth factor (VEGF) transcription and secretion in breast cancer cells. *Oncogene*.

[18] Guo P, Fang Q, Tao H-Q (2003). Overexpression of vascular endothelial growth factor by MCF-7 breast cancer cells promotes estrogen-independent tumor growth in vivo. *Cancer Research*.

[19] Pinto MP, Badtke MM, Dudevoir ML, Harrell JC, Jacobsen BM, Horwitz KB (2010). Vascular endothelial growth factor secreted by activated stroma enhances angiogenesis and hormone-independent growth of estrogen receptor-positive breast cancer. *Cancer Research*.

[20] Marinho A, Soares R, Ferro J, Lacerda M, Schmitt FC (1997). Angiogenesis in breast cancer is related to age but not to other prognostic parameters. *Pathology Research and Practice*.

[21] Weidner N, Semple JP, Welch WR, Folkman J (1991). Tumor angiogenesis and metastasis—correlation in invasive breast carcinoma. *The New England Journal of Medicine*.

[22] Nakamura Y, Yasuoka H, Tsujimoto M (2003). Flt-4-positive vessel density correlates with vascular endothelial growth factor-d expression, nodal status, and prognosis in breast cancer. *Clinical Cancer Research*.

[23] Mumprecht V, Detmar M (2009). Lymphangiogenesis and cancer metastasis. *Journal of Cellular and Molecular Medicine*.

[24] Pavlakis K, Messini I, Vrekoussis T (2008). The assessment of angiogenesis and fibroblastic stromagenesis in hyperplastic and pre-invasive breast lesions. *BMC Cancer*.

[25] Wülfing P, Kersting C, Tio J (2004). Endothelin-1-, endothelin-A-, and endothelin-B-receptor expression is correlated with vascular endothelial growth factor expression and angiogenesis in breast cancer. *Clinical Cancer Research*.

[26] Wülfing P, Tio J, Kersting C (2004). Expression of endothelin-A-receptor predicts unfavourable response to neoadjuvant chemotherapy in locally advanced breast cancer. *British Journal of Cancer*.

[27] Tsutsui S, Yasuda K, Suzuki K, Tahara K, Higashi H, Era S (2005). Macrophage infiltration and its prognostic implications in breast cancer: the relationship with VEGF expression and microvessel density. *Oncology Reports*.

[28] Valković T, Dobrila F, Melato M, Sasso F, Rizzardi C, Jonjić N (2002). Correlation between vascular endothelial growth factor, angiogenesis, and tumor-associated macrophages in invasive ductal breast carcinoma. *Virchows Archiv*.

[29] Chhieng DC, Tabbara SO, Marley EF, Talley LI, Frost AR (2003). Microvessel density and vascular endothelial growth factor expression in infiltrating lobular mammary carcinoma. *Breast Journal*.

[30] Zhou D, Cheng S-Q, Ji H-F (2010). Evaluation of protein pigment epithelium-derived factor (PEDF) and microvessel density (MVD) as prognostic indicators in breast cancer. *Journal of Cancer Research and Clinical Oncology*.

[31] Thomas M, Augustin HG (2009). The role of the angiopoietins in vascular morphogenesis. *Angiogenesis*.

[32] Tsutsui S, Inoue H, Yasuda K (2006). Angiopoietin 2 expression in invasive ductal carcinoma of the breast: its relationship to the VEGF expression and microvessel density. *Breast Cancer Research and Treatment*.

[33] Min Y, Ren X, Vaught DB (2010). Tie2 signaling regulates osteoclastogenesis and osteolytic bone invasion of breast cancer. *Cancer Research*.

[34] Rosen LS, Ashurst HL, Chap L (2010). Targeting signal transduction pathways in metastatic breast cancer: a comprehensive review. *Oncologist*.

[35] Greenberg S, Rugo HS (2010). Triple-negative breast cancer: role of antiangiogenic agents. *Cancer Journal*.

[36] Lee S-H, Mizutani N, Mizutani M (2006). Endoglin (CD105) is a target for an oral DNA vaccine against breast cancer. *Cancer Immunology, Immunotherapy*.

[37] Luo Y, Zhou H, Mizutani M, Mizutani N, Reisfeld RA, Xiang R (2003). Transcription factor Fos-related antigen 1 is an effective target for a breast cancer vaccine. *Proceedings of the National Academy of Sciences of the United States of America*.

[38] Di Cosimo S, Baselga J (2010). Management of breast cancer with targeted agents: importance of heterogenicity. *Nature Reviews Clinical Oncology*.

